# Touchable 3D hierarchically structured polyaniline nanoweb for capture and detection of pathogenic bacteria

**DOI:** 10.1186/s40580-021-00280-9

**Published:** 2021-10-11

**Authors:** Kyung Hoon Kim, MinHo Yang, Younseong Song, Chi Hyun Kim, Young Mee Jung, Nam-Ho Bae, Sung-Jin Chang, Seok Jae Lee, Yong Tae Kim, Bong Gill Choi, Kyoung G. Lee

**Affiliations:** 1 Department of Bioengineering, University of Washington, Seattle, WA 98195-5061 USA; 2 Department of Energy Engineering, Dankook University, Cheonan, 31116 Republic of Korea; 3 Center for Nano Bio Development, National Nanofab Center (NNFC), Daejeon, 34141 Republic of Korea; 4 Department of Chemical and Biomolecular Engineering, Korea Advanced Institute of Science and Technology, Daejeon, 34141 Republic of Korea; 5 Department of Chemistry, Institute for Molecular Science and Fusion Technology, Kangwon National University, Chuncheon, 24341 Republic of Korea; 6 Center for Analysis and Evaluation, National Nanofab Center (NNFC), Daejeon, 34141 Republic of Korea; 7 Department of Chemical Engineering & Biotechnology, Korea Polytechnic University, Siheung-si, 15073 Republic of Korea; 8 Department of Chemical Engineering, Kangwon National University, Samcheok, 25913 Republic of Korea

**Keywords:** Bacteria pathogen, Capture, 3D hierarchical structure, Polyaniline, Polymerase chain reaction

## Abstract

A bacteria-capturing platform is a critical function of accurate, quantitative, and sensitive identification of bacterial pathogens for potential usage in the detection of foodborne diseases. Despite the development of various nanostructures and their surface chemical modification strategies, relative to the principal physical contact propagation of bacterial infections, mechanically robust and nanostructured platforms that are available to capture bacteria remain a significant problem. Here, a three-dimensional (3D) hierarchically structured polyaniline nanoweb film is developed for the efficient capture of bacterial pathogens by hand-touching. This unique nanostructure ensures sufficient mechanical resistance when exposed to compression and shear forces and facilitates the 3D interfacial interactions between bacterial extracellular organelles and polyaniline surfaces. The bacterial pathogens (*Escherichia coli* O157:H7, *Salmonella enteritidis*, and *Staphylococcus aureus*) are efficiently captured through finger-touching, as verified by the polymerase chain reaction (PCR) analysis. Moreover, the real-time PCR results of finger-touched cells on a 3D nanoweb film show a highly sensitive detection of bacteria, which is similar to those of the real-time PCR using cultured cells without the capturing step without any interfering of fluorescence signal and structural deformation during thermal cycling.

**Supplementary Information:**

The online version contains supplementary material available at 10.1186/s40580-021-00280-9.

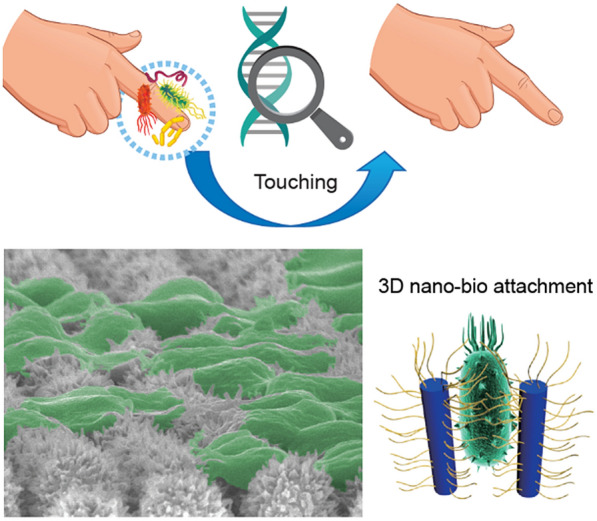

## Introduction

**Fig. 1:**
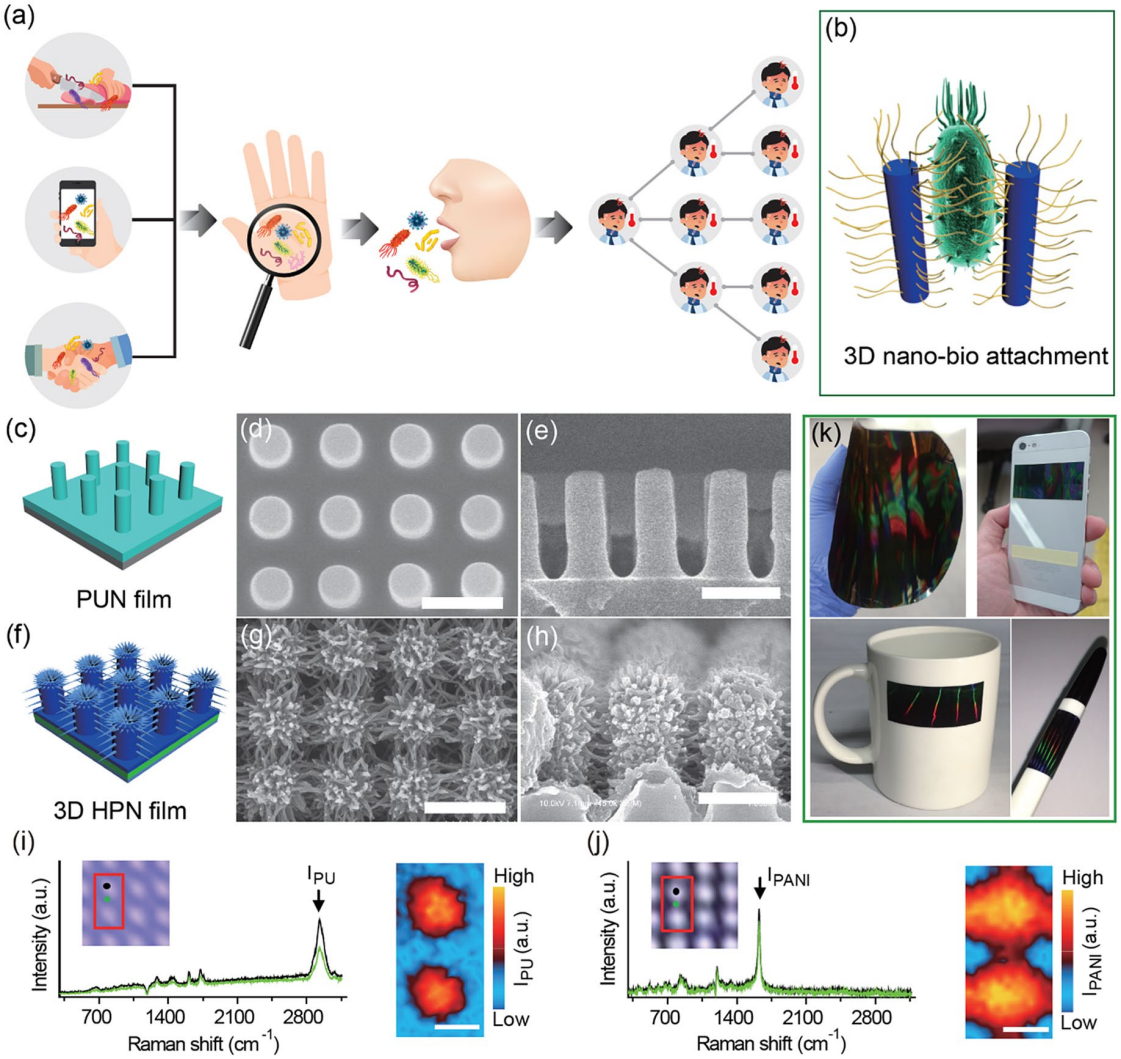
Schematics of **a** a widespread of bacteria pathogens by human hand contacts; **b** a desired attachment of bacteria to a solid surface of capture platform by a pathogen to 3D nanostructural attachments; **c** PUN and **f** 3D HPN films. SEM images of **d**, **e** PUN and **g**, **h** 3D HPN films. Raman spectra and maps of **i** PUN and **j** 3D HPN films. Polyurethane and polyaniline were denoted as PU and PANI, respectively. **k** Photographs of a large-area 3D HPN film and its practical applications as attachments to a smartphone, ceramic cup, and pen. Scale bar: 1 μm

Early-stage detection of pathogenic bacteria, especially foodborne pathogens, becomes a most important task because they cause severe hospitalization and even mortality from foodborne illnesses [1–4]. These diseases are typically spread by everyday used items or food and human-to-human skin contacts (Fig. 1a) [5, 6]. In particular, the most common and critical transmission route of infection propagation becomes by hands [7]. On average, human hands can carry 3200 bacteria from 150 different species, and the World Health Organization (WHO) suggests several guidelines about hygiene to prevent healthcare-associated infections [8–10]. However, most of the diseases have been continuing to occur even though various preventions are applied. Therefore, early diagnostic systems to identify the source of the infection and prevent the local outbreaks for bacterial infectious diseases are required [11].

Up-to-date, great efforts have been made to develop diverse kinds of molecular diagnostics technologies based on the colorimetric [12], light-scattering [13], fluorescence [14], electrochemical [15], and surface-enhanced Raman scattering methods [16]. In these techniques, the accuracy and the sensitivity of the bacterial pathogen identification abilities considerably depend on the effective recovery of pathogenic bacteria from the contaminated inanimate surfaces. However, these efforts are highly motivated to realize the early-stage of diagnosis but it has been rarely investigated to enhance the pathogenic bacterial capturing performance.

More recently, to upgrade the pathogenic bacterial recovery, intensive researches have been carried out to understand the influence of topography on the bacteria and their physicochemical interaction with the structures [17, 18]. In the means of bacterial structure, for instance, when bacteria attach to a solid surface, a complicated attachment process occurs mainly depending on the chemicals that exist around bacteria (e.g., proteins and lipopolysaccharides) [19–21] and the physical structure present on the extracellular organelles (i.e., curli, pili, and flagella fibers) [22–24]. The development of an effective pathogen detection system can, therefore, be achieved when the underlying physicochemical interactions, various nanostructures, and their surface chemical modification strategies are well explored [25]. In particular, one-dimensional nano-array substrates, including silicon nanowires, exhibit an enhanced capture performance by effective topographical interactions with extracellular matrix of bacteria [25–28]. For the hand-caused transmission of bacterial infections, brittle inorganic nanostructured substrates are not suitable for practical bacteria capture by physical contact. Although 1D polymer nanostructure arrays could serve as a soft capture platform [29], the original 1D array structure cannot be maintained when the materials rub the surface of the platform (Additional file 1: Figure S1). The bacteria-capturing platforms with controllable nanostructures, efficient surface modification, and mechanical strength enhancement should, therefore, be continuously developed. More recently, polyainline and zinc oxide have been continuously applied to utilize enhancement of isolation and detection of target biomaterials such as bacteria, nucleic acid, and cells because of their excellent biocompatibility, high aspect ratio, mechanical and chemical stability [30–32]. Furthermore, an in-depth understanding of the attachment of bacteria to the interface of biomolecules and nano-surfaces is required to develop high-performance capture materials [33, 34].

Herein, we report an advanced method to fabricate 3D hierarchically structured polyaniline nanoweb (3D HPN) as an available and efficient pathogenic bacteria-capturing tool with robust mechanical resistance to hand-touching. The critical material design is based on a polyurethane-based nanopillar (PUN) array and secondary growth of polyaniline nanofibers on nanopillars, resulting in a 3D interconnected nanofiber structure. This unique structure ensures a high mechanical resistance when exposed to compression and shear forces, thus enabling bacteria capture through human hand-touching. The complex nanotopographical and physicochemical interaction processes between bacteria and three-dimensional (3D) nanostructures (Fig. 1b) were also intensively investigated. The interfacial interactions between the nano-surface of 3D HPN and bacterial extracellular organelles were intensively investigated by a scanning electron microscope (SEM), a Fourier-transform infrared (FTIR) spectrophotometer, and two-dimensional correlation spectroscopy (2D-COS) analysis. Three different bacteria pathogens of *Escherichia coli* O157:H7 (*E. coli* O157:H7), *Salmonella enteritidis* (*S. enteritidis*), and *Staphylococcus aureus* (*S. aureus*) were effectively captured by the finger-touching method. Moreover, to demonstrate the capturing capability and highly sensitive response to the pathogenic bacteria, we were selected everyday used items such as a cup, pen as the realistic infection model and the real-time PCR-based molecular analysis. The molecular analysis results successfully confirmed that the 3D HPN exhibited a remarkable single bacterial cell-capturing capability and highly sensitive infectious bacterial detection through finger-toughing without any significant structural deformation and fluorescence signal interfering during thermal cycling.

## Materials and methods

### Fabrication of 3D HPN

The 3D HPN films were prepared by synthesis, where polyaniline was grown on the nanopillar arrays of PUN films. The PUN films were immersed in 1 M HClO_4_ solution (70%, OCI), 0.1 M aniline (99.5%, Sigma-Aldrich), 6 mM ammonium persulfate (98%, Sigma-Aldrich), and deionized (DI) water. It was then stored at 3 °C for 24 h. The 3D HPN films were gently washed with DI water and ethanol several times. The specific fabrication method of PUN films was described in Additional file 1.

### Characterization

The morphology and chemical state of the 3D HPN films were characterized by SEM (Hitachi, S4800) and Raman (NT-MDT). To investigate interfacial interactions of 3D HPN and *E. coli* O157:H7, the cells were dropped onto the surface of 3D HPN, and then time-course FTIR spectra were collected using the JASCO FTIR 4600 in the range of 650‒4000 cm^‒1^ with the attenuated total reflectance (ATR) technique. Then, 2D correlation spectra were calculated using the algorithm based on the numerical method developed by Noda and carried out in MATLAB R2019b [35]. PCA was performed using PLS Toolbox Ver. 8.71 (Eigenvector Research, Inc.) for MATLAB.

### Evaluation of mechanical resistance of 3D HPN and PUN

3D HPN and PUN were placed on a microbalance, then pressed with PDMS block (Dow Corning Corporation) for 5 s to measure the weight applied to the surfaces of 3D HPN and PUN. A compressive force was calculated from the weights and surface area of 1 × 1 cm^2^. A shear force was measured by moving the PDMS block horizontally, keeping 3D HPN and PUN being compressed by consistent pressure.

### Bacterial pathogen capture

Mimicking touching and rubbing was performed. Briefly, 100 µL of highly concentrated bacterial solution (10^8^ cells/mL) was doped on the surface of a human hand wearing nitrile lab gloves, and then the 3D HPN surface was touched and rubbed one-directionally with 9.3 kPa by bacteria-doped nitrile glove-wearing finger. Three different areas for each substrate were analyzed with the PCR. To evaluate the capturing capability of the 3D HPN, a known number of *E. coli* O157:H7 (tenfold serially diluted cell numbers from 10^7^ to 10^3^) was dropped on the 3D HPN surface, and incubated in the Petri dishes maintaining humidity to prevent the drying. After 1 h of incubation, the surface of the 3D HPN was gently rinsed with PBS solution and DI water to remove unbounded and loosely bounded bacteria.

### PCR analysis of captured bacterial pathogens

Three different foodborne pathogens, *E. coli* O157:H7 (ATCC 43894), *S. aureus* (ATCC 29213), and *S. enteritidis* (ATCC 13076), were selected to investigate the bacteria-capturing capability of the 3D HPN. Before the capture of bacteria by the 3D HPN, these three pathogens were cultured in LB for 18 h at 37 °C in a shaking incubator, and two different capturing methods were applied: (1) dropping cells onto the 3D HPN and (2) touching the surface with existing cells using the 3D HPN. To identify the cell capturing capability of the 3D HPN, we serially diluted the dropping number of *E. coli* O157:H7 (from 10^7^ to 10^3^) to the 3D HPN. Once the bacteria were trapped onto the 3D HPN, the genomic DNA was extracted using a G-spin™ total DNA extraction kit (iNtRON Biotechnology). About the quantitative DNA analysis of *E. coli* O157:H7, we employed a real-time PCR by targeting the Shiga toxin 2 (*stx2*) gene with specific primers and probes (Additional file 1: Table S3). To verify the pathogen-capturing performance by touching or rubbing the pathogen-covered surface using the 3D HPN, PCR was performed with DNA extracted from the recovered pathogens. The PCR was performed using a GoTaq® DNA polymerase aimed at the *nucA* gene of *S. aureus* and *sefA* gene of *S. enteritidis* with their specific primers (Additional file 1: Table S3). The PCR amplicons were electrophoretically separated to identify their sizes on a 2% agarose gel with RedSafe™ (iNtRON Biotechnology Inc.) in a 1× TAE buffer at 120 V for 15 min. After the PCR amplicon separation, the product bands were observed using a UV transilluminator. The detailed PCR procedure was described in Additional file 1.

## Results and discussion

### Synthesis and bacterial capturability of 3D HPN

The 3D HPN films are fabricated through the controlled growth of polyaniline nanofibers onto nanopillar arrays. A highly ordered nanopillar array film based on the polymeric blend of polyurethane acrylate and NOA63 is prepared using a single-step soft lithography-based replication process (Additional file 1: Figure S2), which is an effective manufacturing method for large-area films. The nanopillar array used in this study has a diameter, height, and center-to-center distance of 500 nm, 1.25 μm, and 1 μm, respectively (Fig. 1c‒e). The use of the dilute polymerization method allows us to control the growth of polyaniline nanofibers onto nanopillars, fully covering the entire PUN arrays (8-in diameter). The nano-scaled and soft-structured polyaniline nanofibers are grown on the top and sidewalls of nanopillars, resulting in the highly ordered 3D hierarchical structure and nanoweb morphology of the 3D HPN film (Fig. 1f‒h). Along with the SEM images, the Raman spectra presented in Fig. 1i and j indicate that spike-shaped polyaniline nanofibers are closely linked with nanopillars to construct a 3D polyaniline nanoweb structure. For the convenient and universal capture and detection of bacterial pathogens, pathogenic adhesive materials should be available to daily used substrates, such as textiles, metals, and polymers. The feasibility of using 3D HPN films with a ceramic cup, a smartphone, and a pen is presented in Fig. 1k.

### Mechanical characterization 3D HPN

**Fig. 2:**
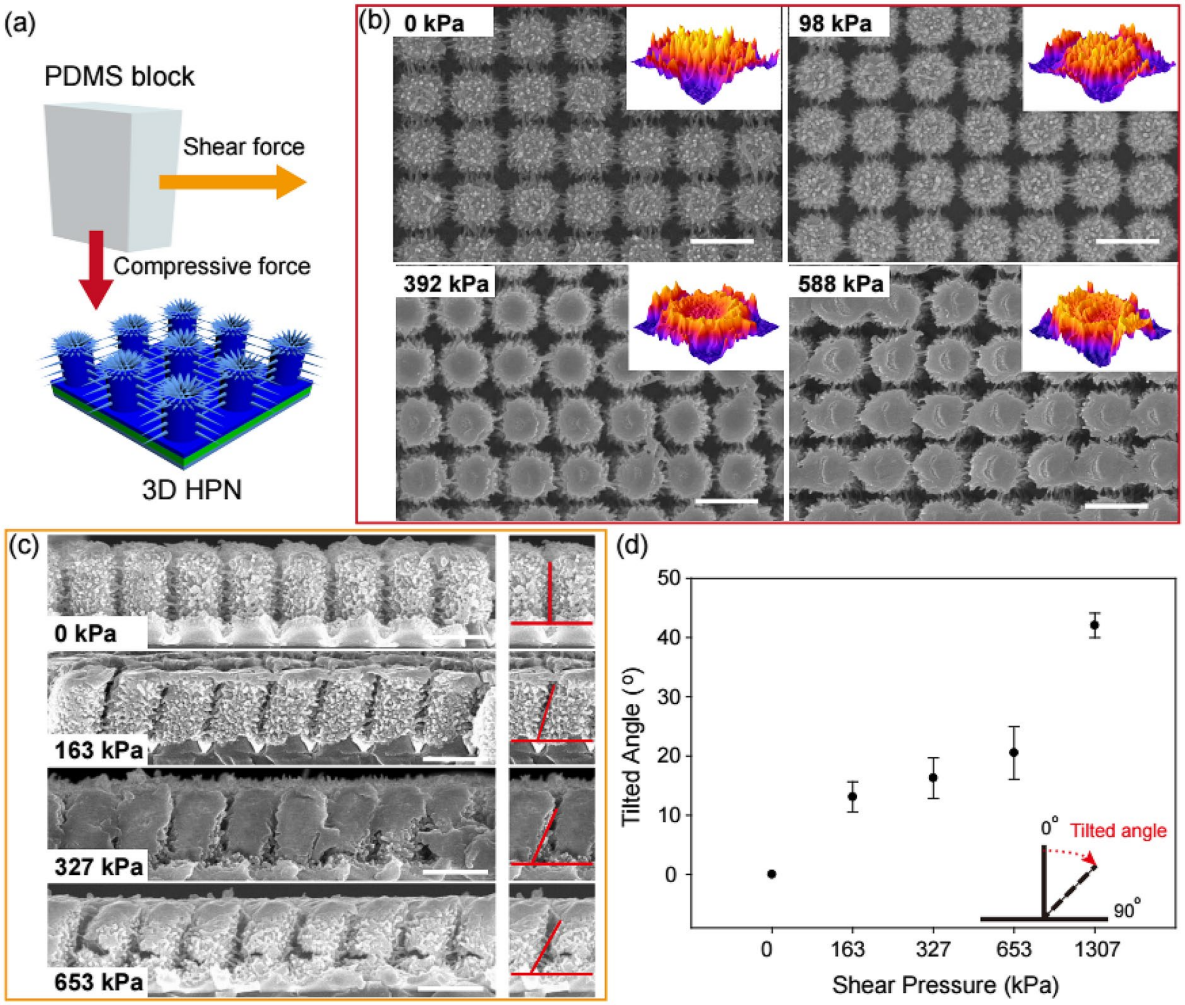
Investigation of the mechanical resistance of the 3D HPN against compressive and shear forces. **a** Schematic of the mechanical test of 3D HPN using the PDMS block. **b** Surface SEM images of the 3D HPN after a 0‒588 kPa compression pressure was applied. Insets are 3D transformed images of corresponding samples, particularly for a single nanopillar. **c** Cross-sectional SEM images of the 3D HPN after a 0‒653 kPa shear pressure was applied. **d** Declined angles of the 3D HPN according to shear pressure. Scale bar is 1 μm

The mechanical resistance of the 3D HPN films to physical contact through finger-touching was investigated by observing the morphological changes of 3D HPN when exposed to compressive and shear forces using a polydimethylsiloxane (PDMS) block (Fig. 2a). By considering that the action of human touching involves pressing and rubbing forces, a typical test of mechanical strength for the touch screen and pressure sensor of nanoscale to microscale substrates is performed in a pressure range of 1‒100 kPa [36–38]. Therefore, the changes in the surface morphology of 3D HPN films are recorded when compressing forces between 0 and 588 kPa (Fig. 2b). It should be noted that the original 3D hierarchical structure remains practically the same, even at a high compressive pressure of 98 kPa. When the pressure further increases to 588 kPa, the nanofibers of polyaniline attached to nanopillars are partially destroyed, but the nanopillar backbone structure of the 3D HPN remains the same (insets in Fig. 2b). The complete collapse of the 3D HPN nanopillar structure is observed at a high compressive pressure of 1961 kPa (Additional file 1: Figure S3a). In contrast, the PUN films with a 1D nanostructure exhibit a severe deformation of nanopillars, which fall off from the film at 98 kPa (Additional file 1: Figure S4).

A shear force is further applied in a direction parallel to the 3D HPN film surface. The structural deformation of 3D HPN when shear forces are applied is observed by the cross-sectional SEM images (Fig. 2c). Besides, the tilted angle of an individual nanopillar from its original position is measured and plotted (Fig. 2d). The 3D HPN nanopillars slightly decline, and the 3D hierarchical structure remains the same when a high shear force of 163 kPa, which fully covers the finger-touching pressure on a screen of a smartphone [29], is applied. As the shear force increases to 653 kPa, the degree of tilted angle also increases (Fig. 2d). The structural deformation of 3D HPN is observed at a significantly high shear stress of 1307 kPa (Additional file 1: Figure S3b). Conversely, at a considerably lower shear stress of 100 kPa, the nanopillars on the PUN film severely deform and agglomerate (Additional file 1: Figure S1). Based on these results, the 3D HPN film is found to have a robust mechanical strength with respect to touching resistance. The enhanced mechanical strength of the 3D HPN compared to that of the 1D nanopillar arrays of the PUN film is attributed to the 3D interconnected polyaniline nanofiber networks.

### Physicochemical interfacial interaction of bacteria and 3D HPN

**Fig. 3:**
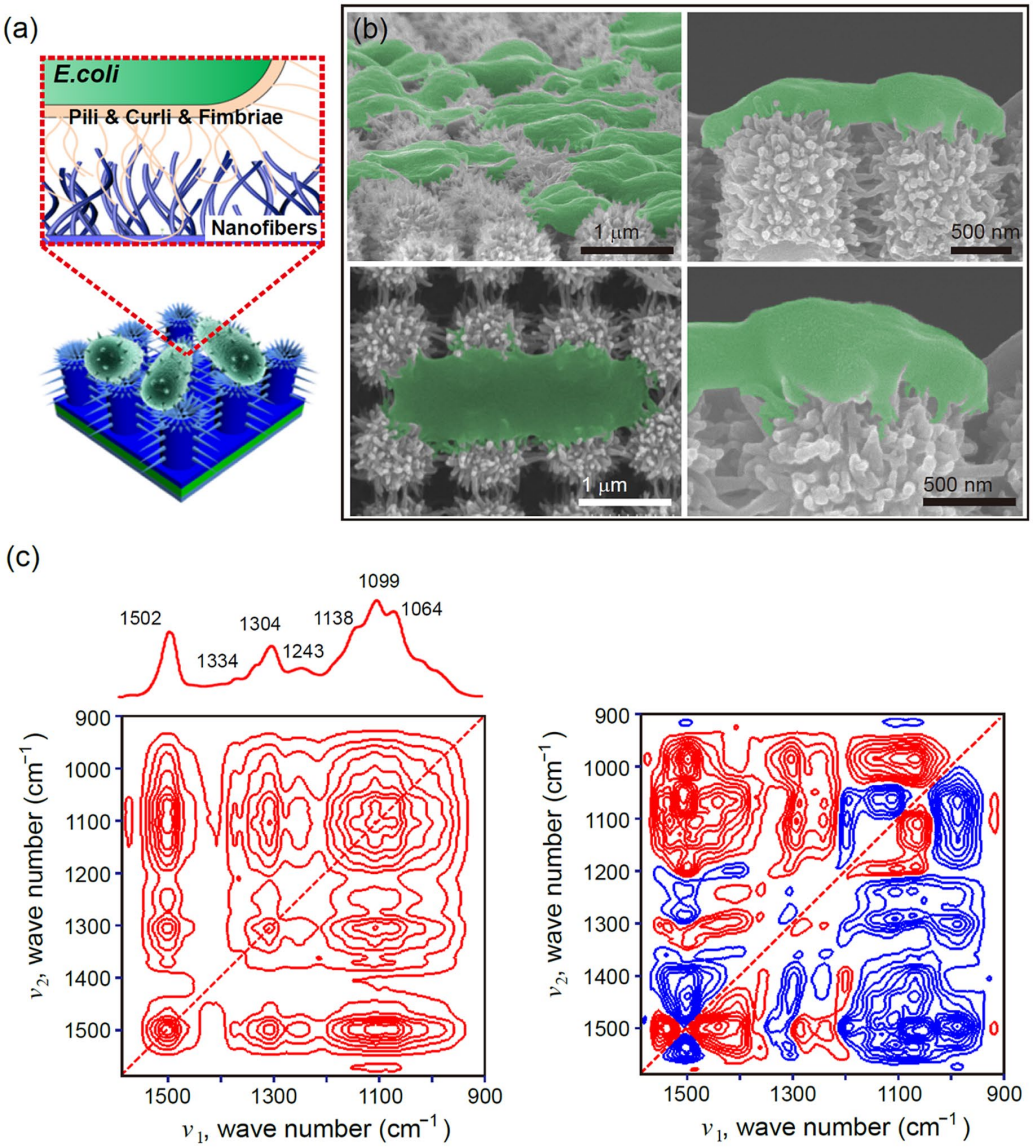
**a** Schematic of interfacial interaction between the bacterial cells and the 3D HPN surface. **b** SEM images of 3D HPN captured *E.coli* O157:H7 cells under different magnifications. **c** Synchronous (left) and asynchronous (right) 2D correlations of the IR spectra of 3D HPN captured *E.coli* cells during *E. coli* O157:H7 adsorption

Understanding the interfacial interaction between the bacteria and 3D HPN film is also highly essential to improve the bacteria-capturing capability of 3D HPN films (Fig. 3a). Since *E. coli* O157:H7 is a well-known infective bacterial pathogen to cause foodborne infections, it was selected in this study [36–38]. The samples of *E. coli* O157:H7 are dropped onto the 3D HPN films and then washed several times with phosphate-buffered saline solution (PBS) and deionized water. The SEM images clearly show that large amounts of bacteria cells are attached to the 3D HPN films (Fig. 3b). The nanostructured extracellular organelles of *E. coli* O157:H7 physically attached to polyaniline nanofibers, resulting in the formation of a bacterial film with a 3D interfacial adhesion. During the bacterial capture process, both pressing and rubbing (i.e., compression and shear forces) are applied to the surface of the 3D HPN. Once the target bacteria were close to the 3D polyaniline nanofibers, the physicochemical forces involved in the adhesion of bacteria to the positively charged 3D polyaniline nanostructure are higher than that of bacteria to the hand or substrates. During this process, the 3D spike-shaped PANI nanofibers enhanced their capturability. In contrast, 1D PUN films show a poor capture platform as bacteria cells are partially attached; the nanopillars are aggregated (Additional file 1: Figure S5).

Apart from physical interactions, microbial adhesion could be simultaneously influenced by the chemically mutual interactions among the surface functional groups of the capture platform and bacteria. Therefore, the chemical interaction at the interface of *E. coli* O157:H7 and 3D HPN was further investigated by observing the chemical changes as a function of adhesion time using an ATR spectroscopy. Then, the spectra were analyzed using the 2D-COS to identify the changes in various functional groups that were not readily provided in the conventional 1D FTIR spectra [42, 43]. The representative functional groups for the polyaniline of 3D HPN and *E. coli* O157:H7 obtained by the ATR measurements are summarized in Additional file 1: Table S1.

Figure 3c shows the synchronous and asynchronous 2D correlation spectra of *E. coli* O157:H7-adsorbed 3D HPN film observed for 24 h. The auto-peaks observed in a diagonal line of the synchronous 2D correlation spectrum indicate the overall intensity changes during *E. coli* O157:H7 adsorption, particularly for the bands at 1502 cm^−1^ (C=C), and significant intensity changes are observed at 1304 and 1138‒1064 cm^−1^ (C‒H and –NH^+^=). In addition, the cross-peak signs are all positive, indicating that the intensity changes of all bands simultaneously increase during *E. coli* O157:H7 binding. The asynchronous 2D correlation spectrum provides the sequential order of intensity changes of bands during *E. coli* O157:H7 adsorption (Fig. 3c, right column). In the asynchronous 2D correlation spectrum, the bands at 1502 and 1304 cm^− 1^ are divided into two bands of 1538 and 1502 cm^− 1^, and 1304 and 1281 cm^− 1^, respectively. 1502, 1281, and 1189 cm^− 1^ are the PANI backbone, and 1538, 1437, 1058, and 1105 cm^− 1^ are bacterial cells. For clarity, all the bands detected in the 2D-COS spectra are listed in Additional file 1: Table S2. The sequence of intensity changes of *E. coli* O157:H7-adsorbed on the 3D HPN is 1437 → 1281 → 1234 → 1538 → 1502 → 1304 → 1058 → 1334 → 1105 → 1133 → 1189 → 981 cm^− 1^. Based on the 2D-COS results, functional moieties of *E. coli* O157:H7 (i.e., C−O−C, C−OH) and PANI (−N=) dominantly contribute to the adsorption of *E. coli* O157:H7 on the surface of 3D HPN. Once again, this indicates that the hydrophobic van der Waals, electrostatic interactions, and hydrogen bonding involve in intermolecular interactions of *E. coli* O157:H7 and the 3D HPN.

### Performance evaluation of 3D HPN for irreversible bacterial capture

**Fig. 4:**
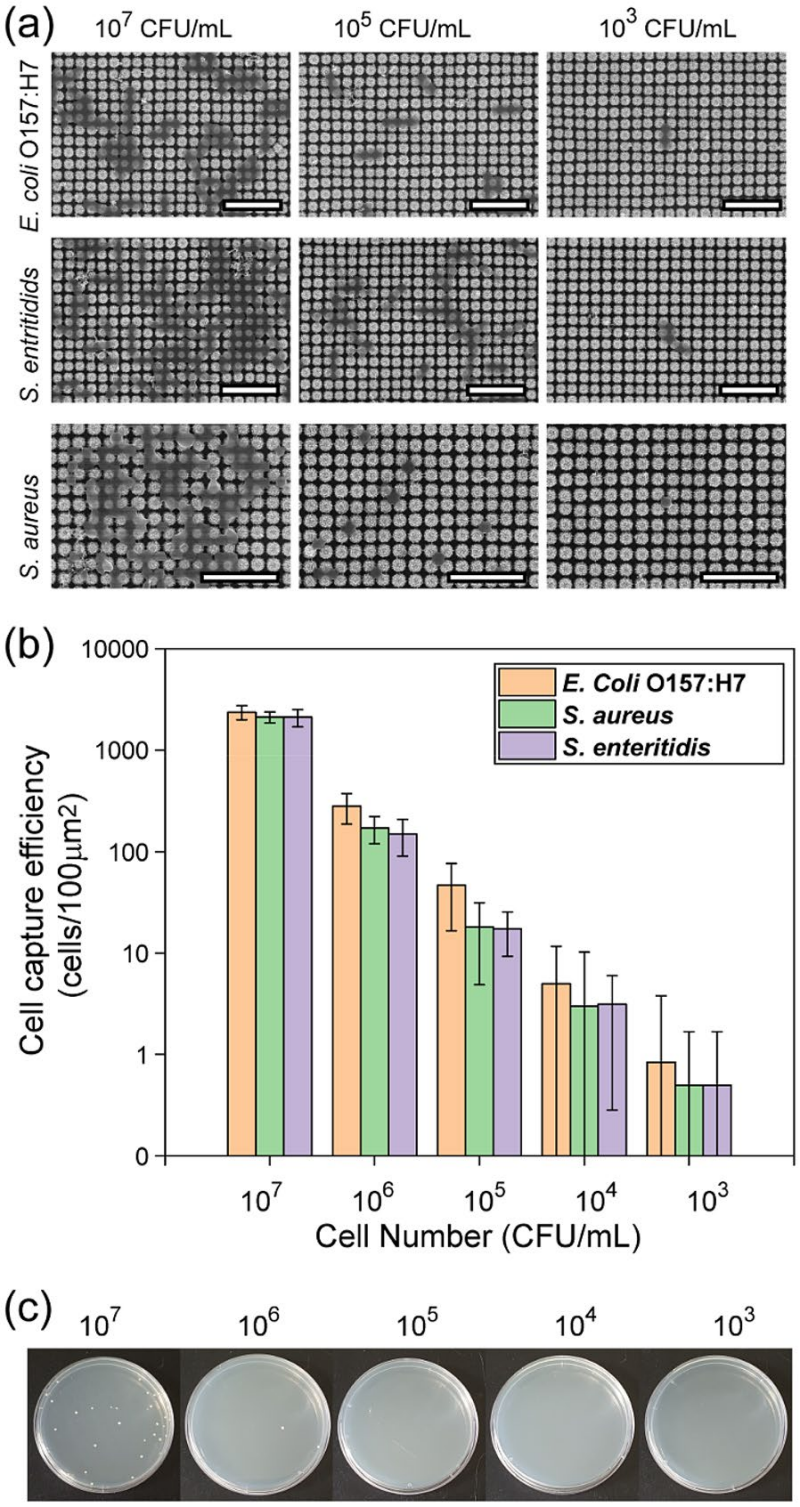
**a** SEM images of 3D HPN captured *E.coli* O157:H7, *S. enteritidis*, and *S. aureus* cells at a cell number of 10^3^_,_ 10^5^_,_ and 10^7^ CFU/mL. Scale bars are 5 μm. **b** Cell capture efficiency results of 3D HPN at a different cell number from 10^3^ to 10^7^ CFU/mL via touching. **c** Representative images of the colony-counting assay results after rubbing *E.coli* O157:H7 captured 3D HPN

To investigate the capture performance of 3D HPN, three most commonly notoriously known Gram-positive and Gram-negative pathogenic bacteria of *E. coli* O157:H7, *S. aureus*, and *S. enteritidis* were carefully selected and tested using 3D HPN. The Gram-positive and Gram-negative bacteria were prepared at different concentrations from 10^3^ to 10^7^ CFU/mL, and 100 µL of each bacteria solution was dropped on the surface of 3D HPN, respectively. The bacteria exposed to 3D HPN was rinsed with DI water to remove uncaptured bacteria. The SEM images of captured bacteria on 3D HPN were shown in Fig. 4a and Additional file 1: Figure S6. Even after instantly touching the bacterial solution using 3D HPN, several bacteria were easily recovered and incorporated on the surface of 3D HPN. As decreasing the amount of the concentration of bacteria, the captured bacteria also proportionally reduced but effectively recover bacteria. In addition, the bacteria fairly maintain their structures and are placed in between the nanopillars. Furthermore, the intact bacteria partially cover the 3D HPN and it is equivalent to the cell numbers of 1 to 2500 per 100 µm^2^ of the 3D HPN after exposure to concentrations of three different bacteria of *E. coli* O157:H7, *S. aureus*, and *S. enteritidis* ranging from 10^3^ to 10^7^ CFU/mL (Fig. 4b).

Irreversible capture of pathogenic bacteria is significantly important to avoid potential secondary infection caused by detachment, transfer, and growing of infectious microbial cells from the surface [44]. To confirm the irreversible capture performance of 3D HPN, the bacteria-captured 3D HPN was rubbed onto the solid Luria broth (LB) agar plates and observed the bacterial colony formation (Fig. 4c). The bacteria-captured 3D HPN were prepared by exposing the *E.coli* O157:H7 solutions at a different concentration from 10^3^ to 10^7^ CFU/mL in the same manner as the bacteria capture test. Then, the bacteria-captured 3D HPN was rubbed to the LB agar plates and cultured overnight. Interestingly, almost no colony (≤ 2 CFU) was formed from the spots even after exposure to the *E. coli* O157:H7 (10^3^ to 10^6^ CFU/mL)-captured 3D HPNs while uncaptured *E. coli* O157:H7 growth on LB agar plates (Additional file 1: Figure S7). Even at a relatively high concentration (10^7^ CFU/mL) that is corresponding to 2,500 CFU/(100 μm)^2^ of bacteria-captured 3D HPN, only 40 CFU were found. In addition, we compared the proposed 3D HPN and cotton swab after exposure to the pathogens and rubbed them to investigate the potential risk through bacterial culture on the LB agar plates. The results showed that after the bacteria-captured cotton swab (exposed 30 s) and 3D HPNs (under different exposure time from 30 s to 60 min) were rubbed to the LB agar plates and cultured overnight. Almost no colony was formed from the spots even after exposure to *E. coli* O157:H7 while the cotton swab shows about 683 colonies (Additional file 1: Figure S8). Based on the result, 3D HPNs exhibited excellent capturability and could avoid potential secondary infection. These results clearly indicate that the 3D HPN can strongly and irreversibly hold the bacteria and prevent potential release to cause secondary infection.

### Potential practical applicability of 3D HPN for pathogenic bacterial capture and detection

**Fig. 5:**
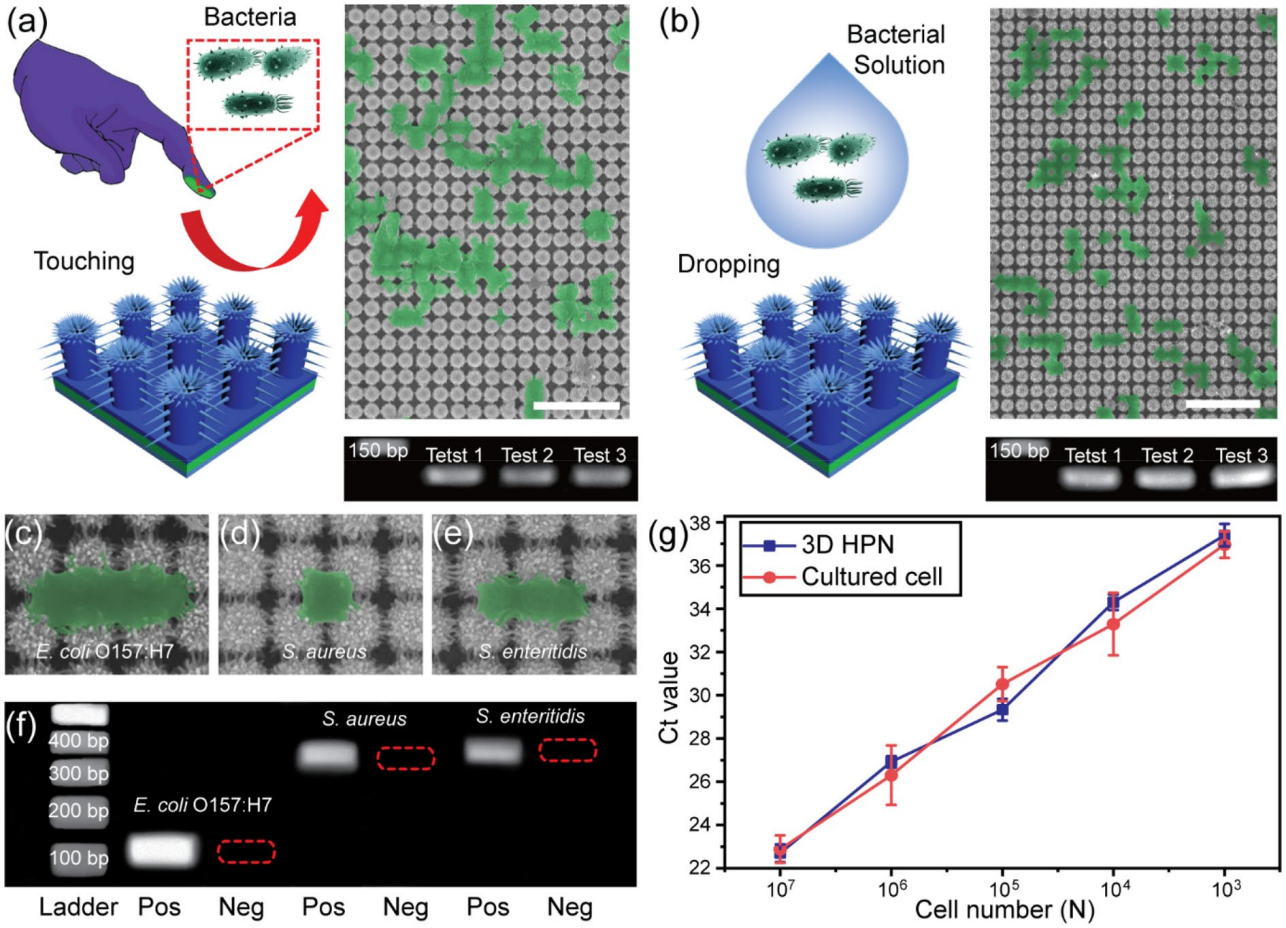
Comparison of bacteria-capturing capabilities of the 3D HPN by **a** touching and **b** dropping method. SEM images showed that *E. coli* O157:H7 cells were attached to the 3D HPN. The genomic DNA of captured *E. coli* O157:H7 on the 3D HPN was amplified in PCR, and amplicons were analyzed by gel electrophoresis. Verification of the versatility of the 3D HPN for three model pathogens covering Gram-positive and Gram-negative bacteria of **c** *E. coli* O157:H7, **d** *S. aureus*, and **e** *S. enteritidis*. **f** Gel electrophoresis results of the three pathogens. **g** Real-time PCR results of captured *E. coli* O157:H7 cells in the finger-touching method and cultured cells in LB broth without recovery step from the 3D HPN at a cell number of 10^3^‒10^7^ CFU/mL

To demonstrate the capability of the 3D HPN films that capture bacteria through finger-touching, the bacterial pathogens captured by the finger-touched films are utilized for further PCR analysis. Here, the conventional capture method where bacterial cells are dropped onto the 3D HPN films without any physical contact is also tested as a control. *E. coli* O157:H7 is cultured in LB medium until the cell population reached 10^9^ cells/mL, and the purified cells are carefully diluted ten times. To evaluate the capacity of bacteria-capturing through touch, a finger, wearing protective latex gloves, dipped into a concentrated bacterial solution (10^8^ cells/mL) and then the surface of 3D HPN (Fig. 5a) is touched with that bacterial-coated finger. After thorough washing, bacteria cells are transferred directly from the glove to the 3D HPN surface, as observed in the SEM image (Fig. 5a). The *stx2* gene of the adsorbed *E.coli* O157:H7 is amplified using the PCR and further investigated by 2.0% agarose gel electrophoresis [45]. As the PCR primers for the *stx2* gene are engineered to synthesize the 121-bp PCR amplicon, the expected bands in three repetitive touching experiments without any non-specific amplification signal are observed. No band is found in the results of the negative controls. The results are consistent with that of the control experiments, where the bacteria are directly dropped onto the 3D HPN at the same concentration of the touching-capture method (Fig. 5b).

In this study, two more pathogens, *S. aureus*, and *S. enteritidis* were further tested for the versatile capture ability of the 3D HPN. They are selected for further analyses as representatives of Gram-negative and Gram-positive bacteria, respectively [46, 47]. These pathogens are also successfully captured on the 3D HPN surface using the finger-touching-capture method. As shown in Fig. 5c–e, these pathogens are strongly attached to the interface of polyaniline nanofibers with a 3D film formation, which is similar to that during *E. coli* O157:H7 adsorption. The PCR analysis and agarose gel electrophoresis exhibit clear product bands of 400 and 498 bp for *S. aureus* and *S. enteritidis*, respectively, and no band signals are observed in the negative controls (Fig. 5f).

To further investigate the bacteria capturing performance of the 3D HPN, real-time PCR analysis was accomplished, since this analytical method provides a rapid, accurate, and quantitative detection of target pathogens by simultaneously measuring the fluorescent signal with amplification. The cell capture is performed by the finger-touching action again within 1 s using the 3D HPN in the *E. coli* O157:H7 concentration range (10^3^‒10^7^ cells/mL). Prior to the real-time PCR analysis, the genomic DNA of the captured pathogen is extracted. As the cell number decreases, the threshold cycle (Ct) values approximately increase linearly from 22.7 to 37.4 (Fig. 5g). It should be noted that these resultant Ct values are similar to those of the obtained real-time PCR results of cultured cells, in which the DNA molecules are directly extracted from cultured cells without the capturing process. Our findings indicate that the excellent bacteria-capturing performance of the 3D HPN is apparent even by using the physical finger-touching process. The thermal stability of the 3D HPN based on the high melting temperature of polyurethane acrylate (PUA, > 170 °C) ensures the PCR cycle in an operating temperature range of 25‒95 °C, no changes in the 3D HPN morphology are observed before and after the PCR analysis (Additional file 1: Figure S9). Additionally, the potential fluorescence interference effects from the 3D HPN during the real-time PCR are verified. The real-time PCR was carried out with/without the 3D HPN and the amplification curves were analyzed using the automated Ct value calculation in the CFX Manager Dx software (Bio-Rad, Berkeley). The Ct values of real-time PCR from the control tube were 24.91 ± 0.045, while the Ct values from 3D HPN including tube were 24.87 ± 1.075 (Additional file 1: Figure S10). These almost no significant Ct changes from both pristine PCR tube and the 3D HPN included tube confirmed the 3D HPN was almost no fluorescent affected to the RT-PCR result.

## Conclusions

In summary, we fabricated 3D hierarchical structured polyaniline nanofiber films available for the touchable-capturing platform of infectious bacterial pathogens. The 3D HPN films exhibited high mechanical resistance to severe external compression and shear forces, resulted from the nanopillar arrays and 3D interconnected polyaniline nanofiber network. Examination of SEM and FTIR spectroscopy measurements revealed that bacterial pathogens were captured onto the surface of 3D HPN by the interfacial interactions. The unique chemical and structural properties of 3D HPN enabled to capture of the bacteria pathogens (i.e., *E. coli* O157:H7, *S. enteritidis*, and *S. aureus*) by the finger-touching method. Based on the real-time PCR results, the 3D HPN by finger-touching exhibited comparable capture performance to the cell culturing method without the capturing step. We believe that the outstanding bacteria-capturing performance of the 3D HPN can be further extended to various bacteria pathogens, thus opening up great opportunities for developing a rapid on-site detecting system of various bacterial pathogens.

## Supplementary Material

**Additional file 1. **Touchable 3D hierarchically structured polyaniline nanoweb for capture and detection of pathogenic bacteria.

## Data Availability

The datasets used and/or analysed during the current study are available from the corresponding authors on reasonable request.
