# Supplementary material for: Touchable 3D hierarchically structured polyaniline nanoweb for capture and detection of pathogenic bacteria
Source: Nano Converg. 2021 Oct 11;8:30. doi: 10.1186/s40580-021-00280-9 (PMC8505581; doi:10.1186/s40580-021-00280-9)
Supplement: Supplementary file 1 — Additional file 1. Touchable 3D hierarchically structured polyaniline nanoweb for capture and detection of pathogenic bacteria. [file 40580_2021_280_MOESM1_ESM.docx]

**Additional Information for**

**Touchable 3D Hierarchically Structured Nanoweb for Capture and Detection of Pathogenic Bacteria**

Kyung Hoon Kim^†,1^, MinHo Yang^†,2^, Younseong Song^3^, Chi Hyun Kim^3^, Young Mee Jung^4^, Nam-Ho Bae^3^, Sung-Jin Chang^5^, Seok Jae Lee^1^, Yong Tae Kim*^,6^, Bong Gill Choi*^,7^,and Kyoung G. Lee*^,3^

^1^Department of Bioengineering, University of Washington, Seattle, WA 98195-5061, United States

^2^Department of Energy Engineering, Dankook University, Cheonan 31116, Republic of Korea

^3^Center for Nano Bio Development, National Nanofab Center (NNFC), Daejeon 34141, Republic of Korea

^4^Department of Chemistry, Institute for Molecular Science and Fusion Technology, Kangwon National University, Chuncheon 24341, Republic of Korea

^5^Center for Analysis and Evaluation, National Nanofab Center (NNFC), Daejeon 34141, Republic of Korea

^6^Department of Chemical Engineering & Biotechnology, Korea Polytechnic University, Siheung-si 15073, Republic of Korea
^7^Department of Chemical Engineering, Kangwon National University, Samcheok 25913, Republic of Korea

1. **Experimental Section**
   1. **Fabrication of Si master mold**

Si wafer was incubated in the furnace (Furnace E1200, Centrotherm) to form a 500 nm SiO_2_ layer. The thermally oxidized wafer was then spin-coated with photoresist to form a layer of 0.7 um thickness. Dot-array patterns with 500 nm of single dot diameter were formed using the KrF scanner (S203-B, Nikon). As-prepared wafers were etched by the ICP (TCP9400SE, Lam Research) with a gas blend of Cl_2_, HBr, and O_2_ to form nanohole arrays.

- 1. **Fabrication of the PUN films:**

PUN films were prepared by spin-coating (Spin coater LSM250, SAWATEC) and the UV polymerizable NOA63 (Norland Optical Adhesives) blended polyurethane (MINS-311RN, Munuta Tech) as a precursor onto the Si nanohole master mold and incubated in the vacuum chamber to remove the entrapped air bubbles. Thin polyethylene (PET) film (Mitsubishi) was placed on the top of the precursor-loaded Si mold. The top PET film was rolled uniformly to eliminate air bubbles and then exposed to the UV illumination (EVG6200, EVG) for 60 s to polymerize and cure the precursor. Consequently, PUN on PET films was peeled off from the master mold.

- 1. **Preparation of the pathogen cells and PCR reagents:**

*E. coli* O157:H7 (ATCC 43894), *S. aureus* (ATCC 29213), and *S. enteritidis* (ATCC 13076) were purchased from the Korean Culture Center of Microorganisms. The pathogens were cultivated in 5 mL of LB broth (LPA solution) at 37 °C in a shaking incubator at 150 rpm for 18 h, and then the cultivated bacteria were thoroughly washed using 0.1 M PBS. To verify the bacteria-capturing ability of the 3D HPN, different capturing methods, such as dropping cells onto the 3D HPN and rubbing cell-existed surface using the 3D HPN, were utilized.

For the genetic analysis for the captured cells by the 3D HPN, the genomic DNAs of the three pathogens were extracted using the G-spin^TM^ total DNA extraction kit (iNtRON Biotechnology) following the manufacturer’s protocol. Real-time PCR analyses were carried out to investigate the 3D HPN capturing capability of *E. coli* O157:H7 by targeting the *stx2* gene. A reaction volume (25 μL) of PCR mixture included 12.5 μL of 2× AmpliTaq Gold^®^ Fast PCR Master Mix (Applied Biosystems), 2 μL of 150 nM of forward and reverse primers, and 65 nM TaqMan probe mixture (forward primer: 5’-GGG CAG TTA TTT TGC TGT GGA-3’, reverse primer: 5’-TGT TGC CGT ATT AAC GAA CCC-3’ and TaqMan probe: 5’-FAM-CTA TCA GGC GCG TTT TGA CCA TCT TCG-TAMRA-3'), and 1 μL of genomic DNA.

To identify the cell capturing capability of the 3D HPN, cultured bacterial cell solutions were serially diluted and dropped onto the 3D HPN. Genomic DNA was then extracted and used for real-time PCR analyses. The thermal cycling process was carried out using the AriaMx Real-Time PCR System (Agilent Technologies) with the following protocol: an initial activation at 95 °C for 10 min, 40 cycles of 95 °C for 5 s, 60 °C for 5 s, 68 °C for 10 s, and a final extension step at 72 °C for 10 s. The fluorescence signal was detected from every extension step during the thermal cycling.

The conventional PCR was performed using the genomic DNA, which was extracted from the three pathogens that were collected by touching and rubbing or dropping methods. The DNA amplification was performed using the GoTaq^®^ DNA Polymerase for the *nucA* gene of *S. aureus* and *sefA* gene of *S. enteritidis*. 25 μL of PCR mixture was composed of 5 μL of 5× GoTaq^®^ Reaction Buffer, 1.25 μL of GoTaq^®^ DNA Polymerase (5 U/μL), 2 μL of 10 mM dNTP, 2 μL of 10 μM forward and reverse primers, and 1 μL of genomic DNA. The primer sequences of the target pathogens are as follow: *S. aureus* (forward primer: 5’- AAA GCG ATT GAT GGT GAT ACT GT-3’, and reverse primer: 5’-GAC CTG AAT CAG CGT TGT CTT CG-3’), *S. enteritidis* (forward primer: 5’- CAA AGC AGT GGT TCA GGC AG-3’, and reverse primer: 5’- TGC TGA ACG TAG AAG GTC GC-3’). To amplify the DNA of *E. coli* O157:H7, the same primers, which were used in the real-time PCR, were utilized. The PCR was accompanied with the DNA Engine Thermal Cycler (Bio-Rad, Hercules, CA) by the following protocol: an initial activation at 95 °C for 5 min, 30 cycles of 95 °C for 30 s, 60 °C for 30 s, 72 °C for 30 s, and a final extension step at 72 °C for 5 min. The PCR products were then confirmed by gel electrophoresis, which was carried out on 2% agarose gel with RedSafe^TM^ (iNtRON Biotechnology Inc.) in a 1 × TAE buffer at 120 V for 15 min. After the separation of the PCR amplicons, the product bands were observed using a UV transilluminator.

**1.4. Fluorescence Signal interfering testing of the 3D HPN**

*E. coli* O157:H7 was cultured at 37 °C for 18 h in 10 mL of a Luria–Bertani (LB) broth containing 1 g of sodium chloride, 0.5 g of yeast extract, and 1 g of tryptone in 100 mL of autoclaved water. The cultured cell number in the broth was calculated using the colony counting method. The suspended *E. coli* O157:H7 was spiked into a broth and then diluted to obtain 10^6^ CFU of *E. coli* O157:H7 per 100 µL broth. *E. coli* O157:H7 bacterial sample was mixed with 100 µL QuickExtract^TM^ DNA extraction solution 1.0 (Lucigen) in 1.5 mL microcentrifuge tube, and incubated at 98 °C for 2 min as a pre-treatment of bacteria lysis.

Real-time PCR was carried out with extracted DNAs from *E.coli* O157:H7 to investigate the optical interference of the 3D HPN. The 25 µL of real-time PCR cocktail was composed of 1 µL of extracted genomic DNA, 12.5 µL of 2X Amplitaq Gold Fast Master Mix (Applied Biosystems, Foster City, CA), 2 µL of 150 nM forward and reverse primers, and 65 nM TaqMan probe mixture (forward primer: 5’-GGG CAG TTA TTT TGC TGT GGA-3’, reverse primer: 5’-TGT TGC CGT ATT AAC GAA CCC-3’ and TaqMan probe: 5’-FAM-CTA TCA GGC GCG TTT TGA CCA TCT TCG-TAMRA-3’). Thermal cycling condition for real-time PCR consists of 95 °C for 10 min for initial denaturation, 40 cycles of 96 °C for 5 sec, 60 °C for 5 sec, 68 °C for 10 sec. The real-time PCR was performed using CFX 96^TM^ (Bio-Rad, Berkeley) and the results were analyzed using the automated threshold cycle (Ct) value calculation in the CFX Manager Dx software (Bio-Rad). For fluorescence signal interfering test of the 3D HPN, 1.5 mm X 1.5 mm sized 3D HPN was inserted inside of the PCR tube.

**Table S1.** FTIR results of the 3D HPN and *E. coli* O157:H7.

|  | Wavenumber (cm^−1^) | Assignment [1,2] |
| --- | --- | --- |
| 3D HPN | 1556 | C=C stretching vibration of quinonoid |
|  | 1482 | C=C stretching vibration of benzenoid |
|  | 1293 | C−N stretching vibration of secondary aromatic amine |
|  | 1247 | C−N^•+^ stretching vibration in benzenoid |
|  | 1132 | C−H in-plane bending vibration of the aromatic ring  –NH^+^= stretching vibration |
|  | 820 | C−H out of plane deformation |
|  | 623 | C−H out of plane bending vibration |
| *E. coli* O157:H7 | 3298 | N−H stretching and O−H stretching vibration |
|  | 2965 | C−H stretching (asymmetric) vibration of –CH_3_ in fatty acids |
|  | 1655 | Amide I |
|  | 1543 | Amide II |
|  | 1402 | C=O stretching (symmetric) of COO^−^ of proteins |
|  | 1239 | P=O stretching (asymmetric) vibration |
|  | 1200 − 800 | C−O−C of polysaccharide and stretching vibration of phosphate |
|  | 1076 | C−OH stretching of oligosaccharide |

**Table S2.** FTIR band assignments of the 3D HPN while *E. coli* O157:H7 adsorped considering the 2D-COS analysis.

| Wavenumber (cm) | Assignments [1–3] |
| --- | --- |
| 1538 | Amide II |
| 1502 | C=C stretching vibration of benzenoid |
| 1437 | C−H deformation of >CH_2_ of proteins |
| 1281 | C−N stretching vibration of secondary aromatic amine |
| 1189 | C−H bending vibration of the aromatic ring |
| 1105 | C−O−C of polysaccharide |
| 1058 | C−OH stretching of oligosaccharide |
| 981 | C−H out of plane deformation |
| 838 | C−H out of plane deformation |

**Table S3**. Sequences of the PCR primers.

| Number | Bacterial strain | Sequences of primers and probe (5‘-3‘) | | Target gene | Amplicon size (bp) |  |
| --- | --- | --- | --- | --- | --- | --- |
| 1 | *E. coli* O157:H7 | FWD | GGG CAG TTA TTT TGC TGT GGA | *stx2* | 121 | |
|  |  | REV | TGT TGC CGT ATT AAC GAA CCC |  |  |  |
|  |  | Probe | FAM-CTA TCA GGC GCG TTT TGA CCA TCT TCG-TAMRA |  |  |  |
| 2 | *S. aureus* | FWD | AAA GCG ATT GAT GGT GAT ACT GT | *nucA* | 400 | |
|  |  | REV | GAC CTG AAT CAG CGT TGT CTT CG |  |  |  |
| 3 | *S. enteritidis* | FWD | CAA AGC AGT GGT TCA GGC AG | *sefA* | 498 | |
|  |  | REV | TGC TGA ACG TAG AAG GTC GC |  |  |  |


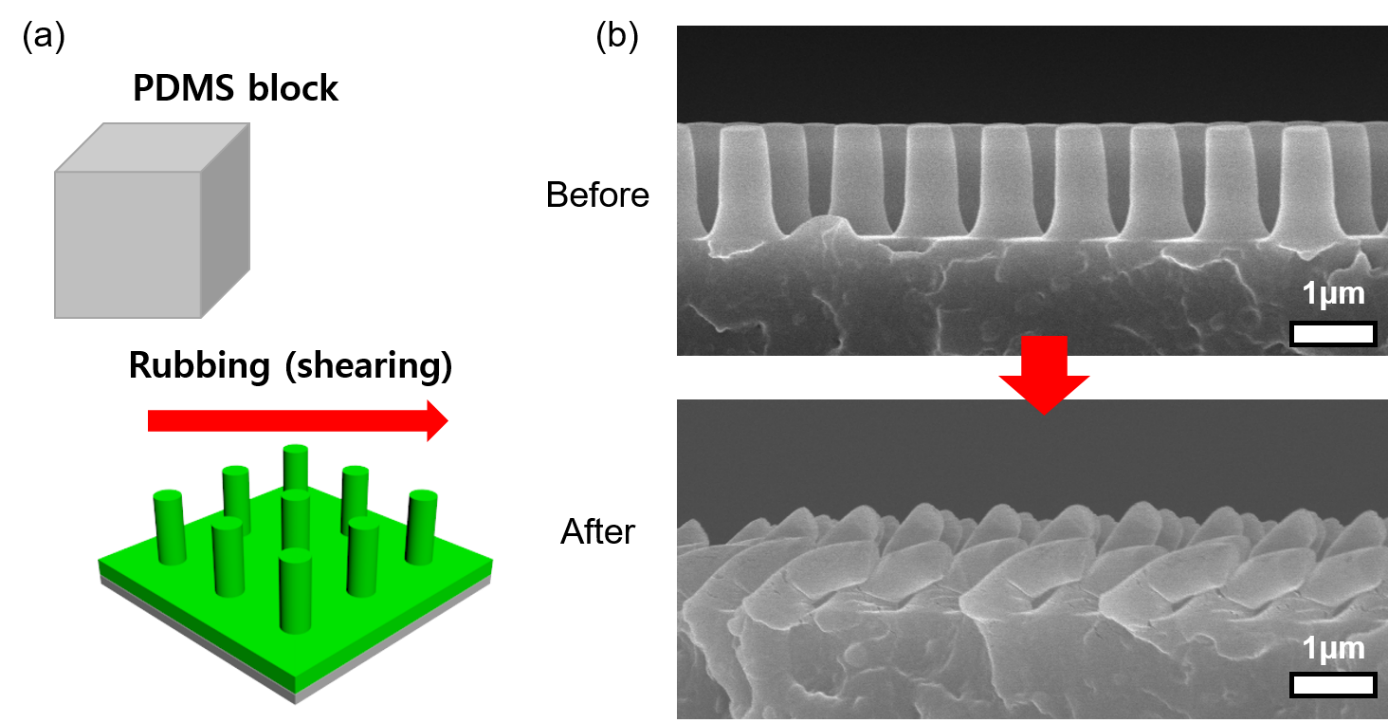


**Figure S1**. (a) Schematic illustration of the PUN arrays after exposure to an external shear force. (b) Cross-sectional SEM images of the PUN film before and after applying 100 kPa shear force using the polydimethylsiloxane (PDMS) block.


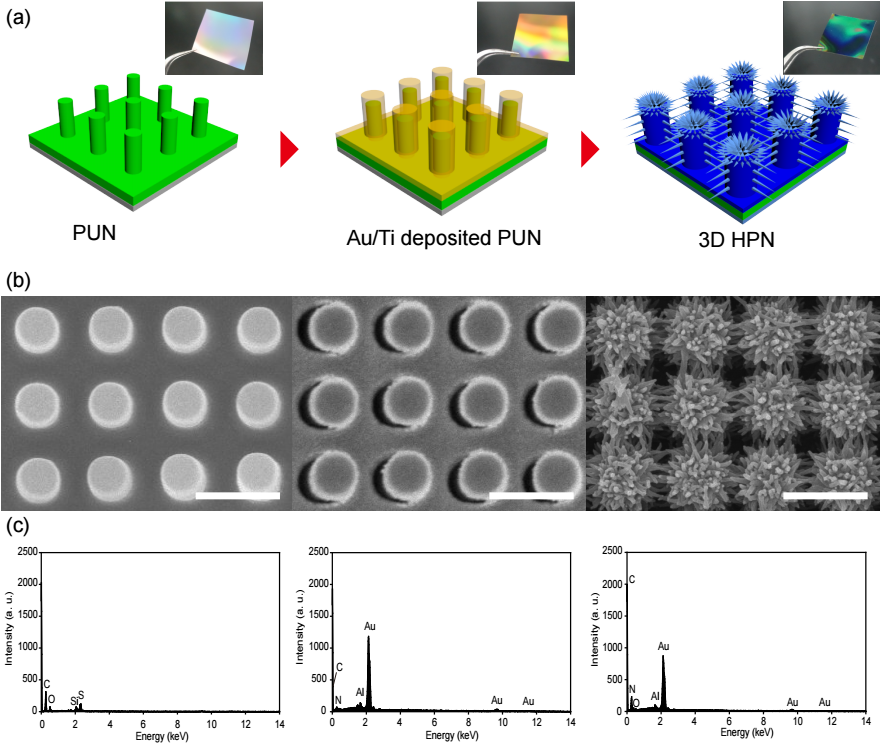


**Figure S2**. (a) Schematic illustration and photographs for the fabrication of the 3D HPN films. (b) SEM images and (c) EDX spectra of the PUN nanopillar array, Au/Ti-coated PUN, and 3D HPN, respectively.


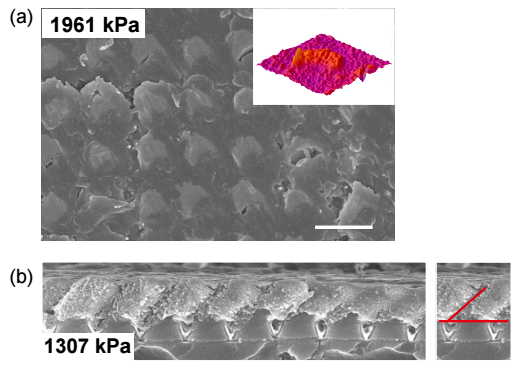


**Figure S3**. (a) SEM image of the 3D HPN surface after applying 1961 kPa of compression pressure. Inset is a 3D transformed image of a single nanopillar. (b) Cross-sectional SEM image of the 3D HPN after applying 1307 kPa of shear pressure. Scale bar: 1 μm.


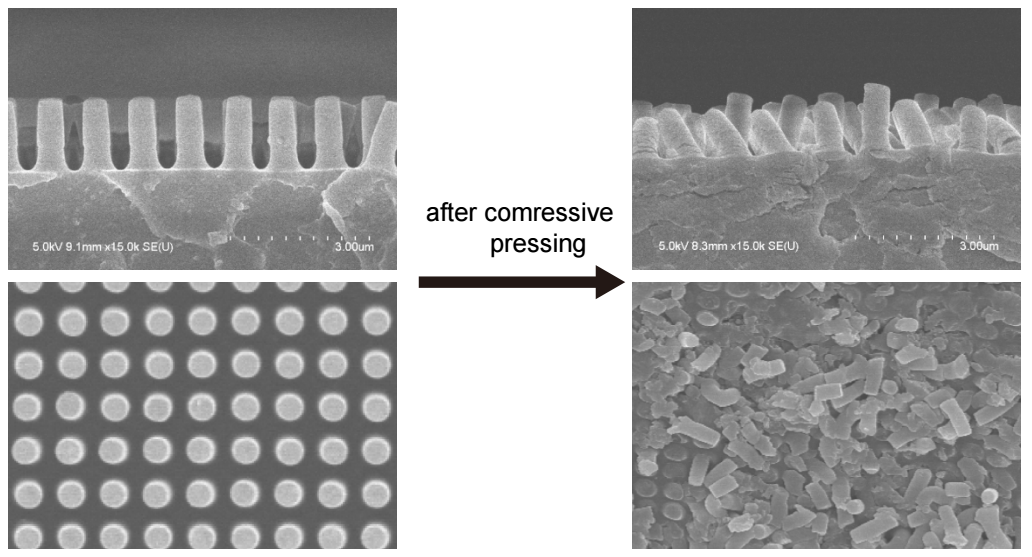


**Figure S4**. SEM images of the PUN film before (left) and after (right) compressive pressure of 98 kPa.





**Figure S5**. SEM image of the *E. coli* O157:H7 solution dropped PUN film.

**
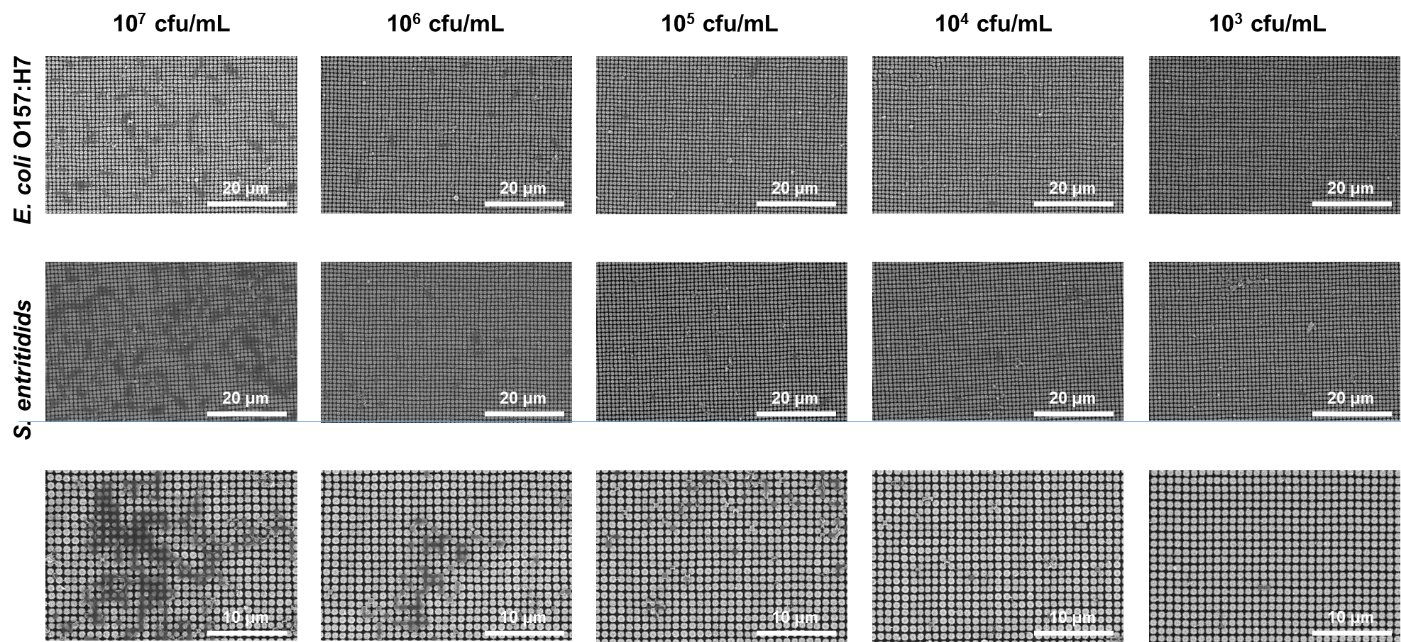
**

**Figure S6**. SEM images of the 3D HPN after exposed to the three different pathogenic bacterial concentration from 10^3^ to 10^7^ CFU/mL.


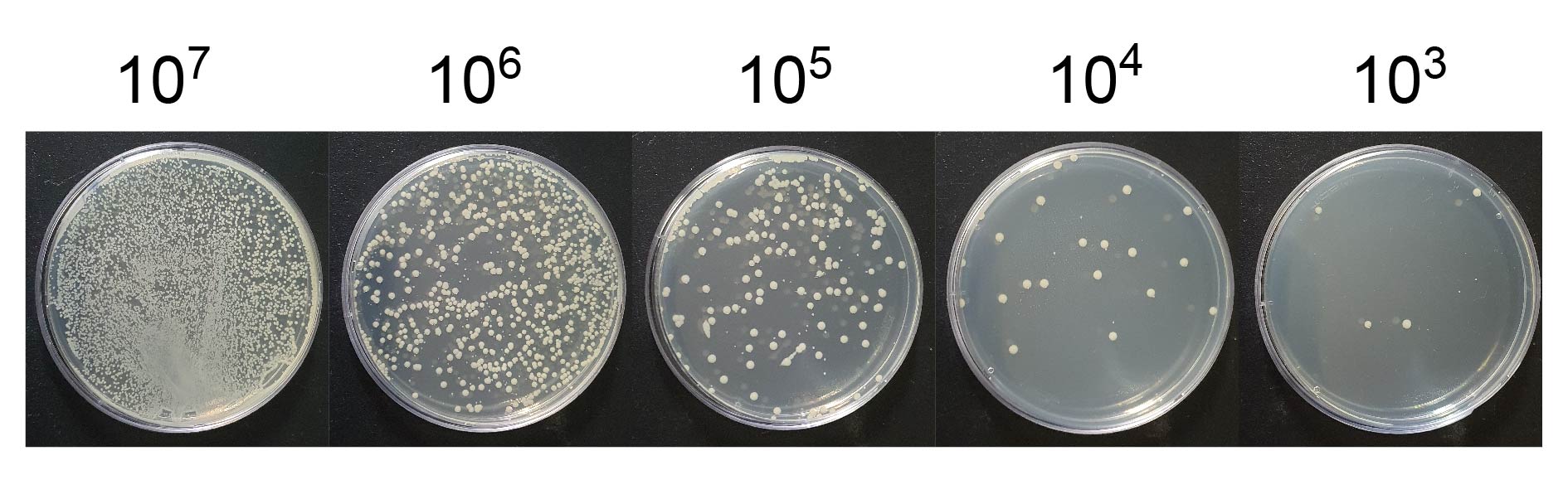


**Figure S7**. Representative images of the colony‐counting assay results with different bacterial concentration from 10^3^ to 10^7^ CFU/mL of *E. coli* O157:H7 growth on LB agar plates.


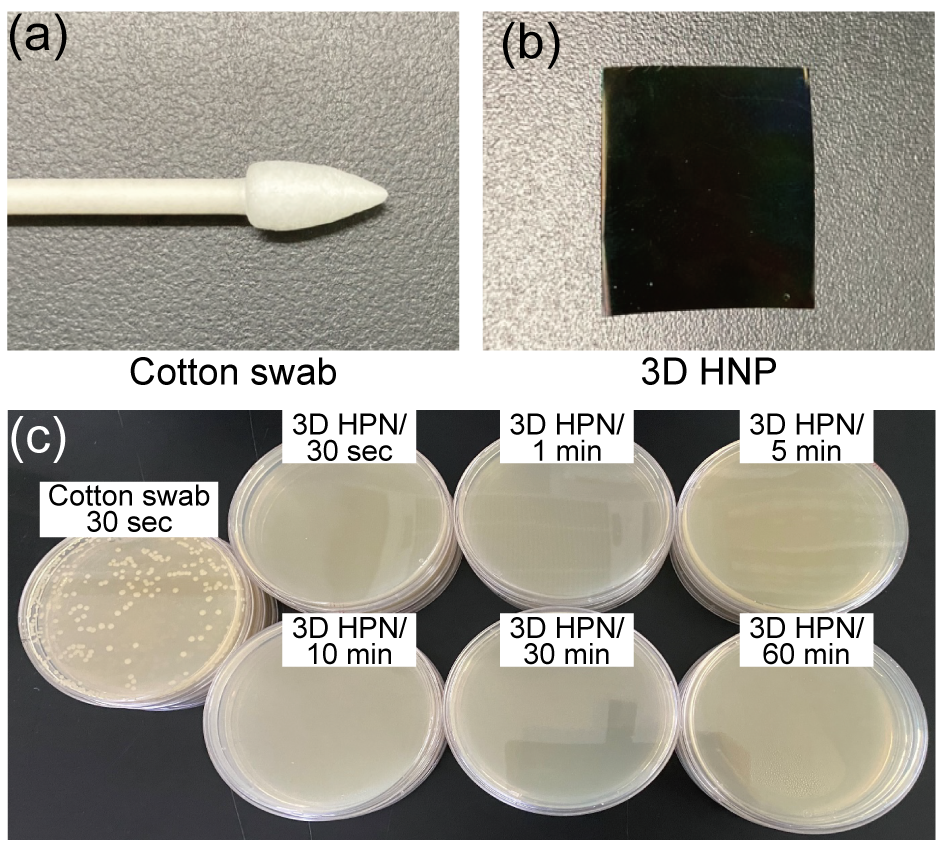


**Figure S8**. Photograph of (a) cotton swab and (b) 3D HPN. (c) *E. Coli* O157:H7 cell culture on LB agar using both cotton swab and 3D HPNs under different exposure time.


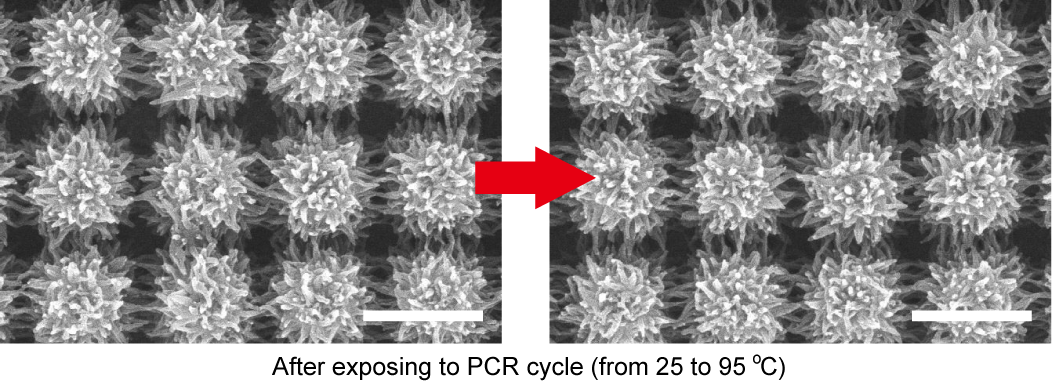


**Figure S9**. Verification of thermal stability of the 3D HPN. SEM images of the 3D HPN before (left) and after (right) PCR cycles at a temperature of 95^o^C. Scale bars are 1 μm.


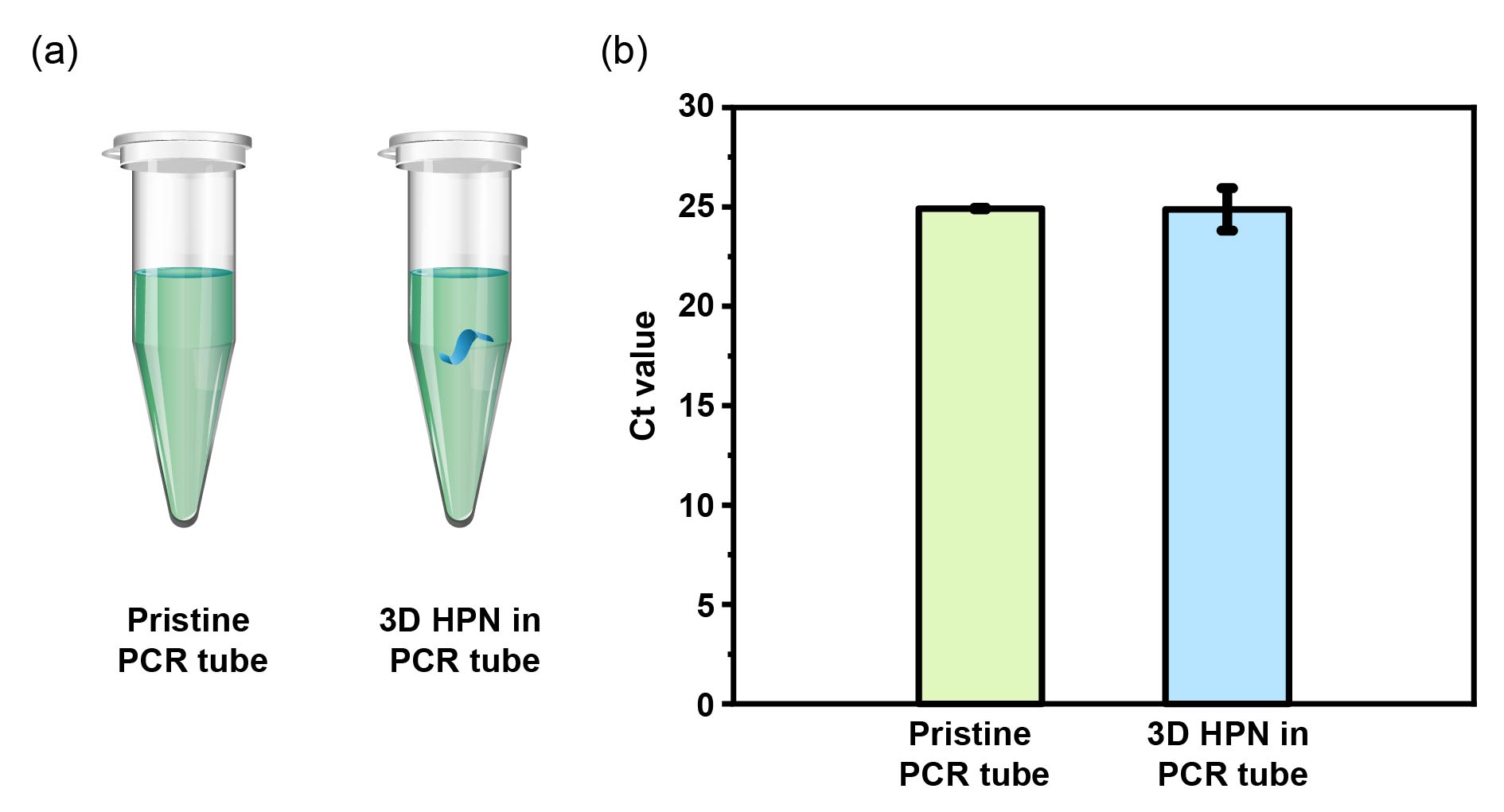


**Figure S10**. (a) Schematic illustration of pristine PCR tube with and without 3D HPN. (b) Ct value of pristine PCR tube with and without 3D HPN.

**References**

1. M. Trchová, I. Šeděnková, E. Tobolková, and J. Stejskal, Polym. Degrad. Stab. **86**, 179 (2004).

2. X. Lu, Q. Liu, D. Wu, H. M. Al-Qadiri, N. I. Al-Alami, D. H. Kang, J. H. Shin, J. Tang, J. M. F. Jabal, E. D. Aston, and B. A. Rasco, Food Microbiol. **28**, 537 (2011).

3. D. Naumann, G. Barnickel, H. Bradaczek, H. Labischinski, and P. Giesbrecht, Eur. J. Biochem. **125**, 505 (1982).
